# Supplementary material for: A novel microglial subset plays a key role in myelinogenesis in developing brain
Source: EMBO J. 2017 Sep 28;36(22):3292–308. doi: 10.15252/embj.201696056 (PMC5686552; doi:10.15252/embj.201696056)
Supplement: Supplementary file 1 — Expanded View Figures PDF [file EMBJ-36-3292-s001.pdf]

Expanded View Figures

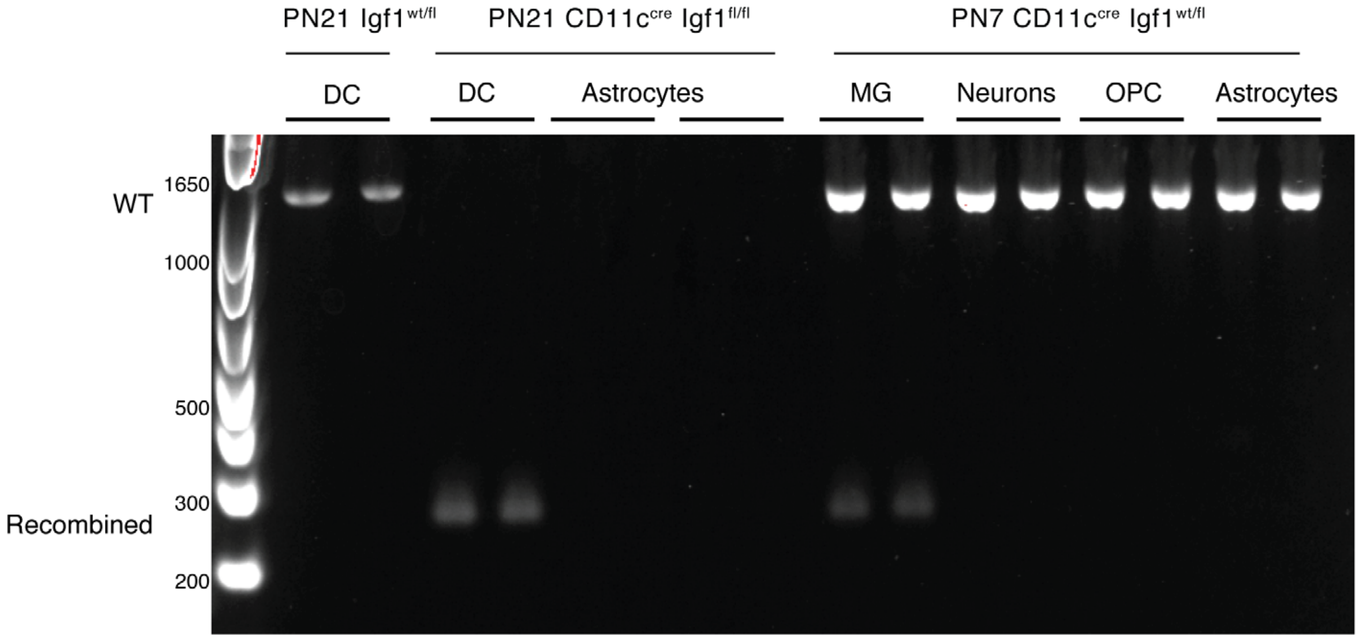

Figure EV1. Cre recombination in microglia.

Genomic PCR analysis of Cre recombination in MACS-sorted splenic dendritic cells (DC) from PN21 Igf1$^{wt/fl}$; DC, astrocytes from PN21 CD11c$^{Cre\text{-}GFP}$ Igf1$^{fl/fl}$ (homozygous) as well as microglia, neurons, OPC, and astrocytes from CD11c$^{Cre\text{-}GFP}$ Igf1$^{wt/fl}$ (heterozygous). Wild-type *Igf1* gene is detected as an ~1-kb band; Cre-induced recombination is detected as an ~0.2-kb band, while the Igf1/flox locus cannot be amplified under the assay condition (Liu *et al*, 1998).

 © 2017 The Authors
