# Supplementary figures and images for: A novel microglial subset plays a key role in myelinogenesis in developing brain
Source: EMBO J. 2017 Sep 28;36(22):3292–308. doi: 10.15252/embj.201696056 (PMC5686552; doi:10.15252/embj.201696056)

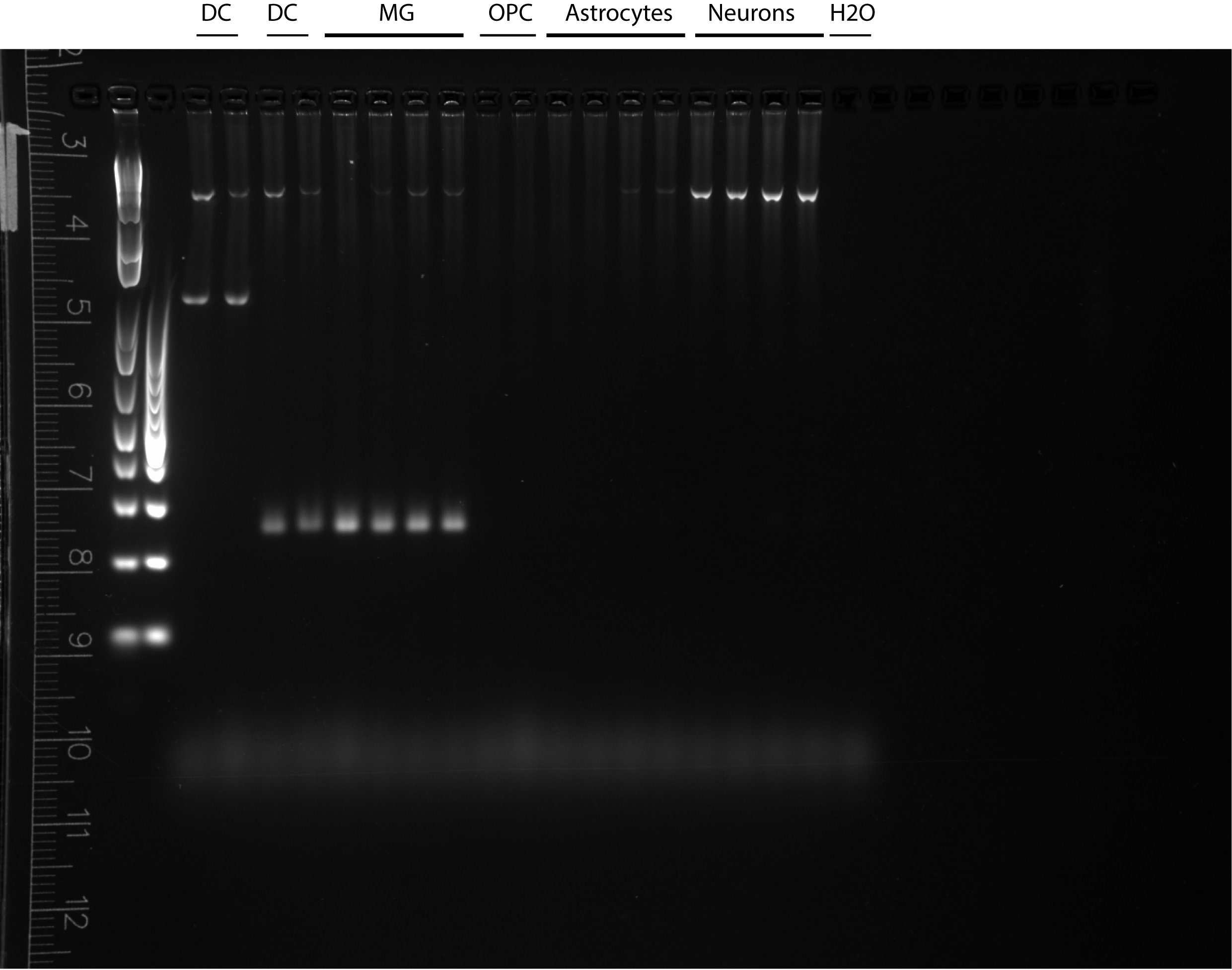

Supplement: Supplementary file 4 — Source Data for Figure 2 [file EMBJ-36-3292-s003.jpg]
